# Outcomes for Patients with Diabetic Foot Ulcers Following Transition from Medicaid to Commercial Insurance ^†^

**DOI:** 10.3390/diabetology5030027

**Published:** 2024-08-21

**Authors:** KiBeom Kwon, Taylor A. Brown, Juan C. Arias Aristizábal, David G. Armstrong, Tze-Woei Tan

**Affiliations:** 1 Elson S. Floyd College of Medicine, Washington State University, Spokane, WA 99202, USA; 2 College of Medicine, University of Arizona, Tucson, AZ 85724, USA; 3 Keck School of Medicine, University of Southern California, Los Angeles, CA 90033, USA

**Keywords:** Medicaid, commercial insurance, diabetic foot ulcer, major amputation

## Abstract

**Objective:**

This study investigates the outcomes of Medicaid beneficiaries with diabetic foot ulcers (DFUs) who had transitioned to commercial insurance.

**Methods:**

We utilized the PearlDiver claims database to identify adult patients diagnosed with a new DFU between 2010 and 2019. The study cohort comprised 8856 Medicaid beneficiaries who had at least three years of continuous enrollment after DFU diagnosis. Medicaid beneficiaries who transitioned to Medicare during follow-up were excluded. Adjusted comparisons of outcomes were performed by propensity matching the two groups for age, gender, and Charlson Comorbidity Index (CCI) in a 1:1 ratio. We used logistic regression and Kaplan–Meier estimate to evaluate the association between insurance change (from Medicaid to commercial insurance) and major amputation.

**Results:**

Among the 8856 Medicaid beneficiaries with DFUs, 66% (*n* = 5809) had transitioned to commercial insurance coverage during follow-up. The overall major amputation rate was 2.8% (*n* = 247), with a lower rate observed in patients who transitioned to commercial insurance compared to those with continuous Medicaid coverage (2.6% vs. 3.2%, *p* < 0.05). In multivariable analysis, Medicaid beneficiaries who transitioned to commercial insurance had a 27% lower risk of major amputation (study cohort: odds ratios [OR] 0.75, 95% CI 0.56–0.97, *p* = 0.03; matched cohort: OR 0.65, 95% 0.22, 0.55, *p* = 0.01) compared to those with continuous Medicaid coverage.

**Conclusions:**

Transitioning from Medicaid to commercial insurance may be associated with a lower risk of major amputation among Medicaid beneficiaries with DFUs.

## 1. Introduction

Foot ulceration is a significant complication among individuals with diabetes, resulting from underlying neuropathy, poor glycemic control, peripheral artery disease (PAD), and other comorbidities [1,2]. The risk of developing a diabetic foot ulcer (DFU) over the lifetime of person with diabetes is between 19% to 34% [1], with an additional risk of lower limb amputation ranging between 10% to 15% after onset of an initial ulcer [3,4]. DFUs are the leading cause of non-traumatic amputations among Americans with diabetes, resulting in annual medical costs exceeding $9 billion, along with loss of productivity [5,6].

The literature on health disparities in DFU and limb loss has been well documented. Studies have consistently found that patients identifying as Black and those residing in rural and lower-income neighborhoods are at an increased risk of amputation secondary to diabetes and PAD [7–12]. Socioeconomic factors, such as low median household income, Medicaid insurance, and uninsured status, are associated with a greater risk of amputation and a lower likelihood of undergoing limb salvage revascularization [7,11,13–16]. Furthermore, among patients with DFUs, socioeconomic disadvantage is identified as an independent predictor of mortality, even after adjusting for baseline age, gender, and comorbidities [17].

Health insurance status is an important factor that may influence patients’ access to care and treatment for diabetes, including the management of DFUs. Studies have shown that having any health insurance, including Medicaid, is associated with better glycemic control among individuals with diabetes compared to those who are uninsured [18]. Furthermore, racial and ethnic minority adults with DFUs in states that expanded their Medicaid programs under the Affordable Care Act (ACA) in 2012 have been found to experience decreased rates of major amputation, compared to those in non-expanded states [19]. While previous research has indicated a higher rate of amputation among Medicaid beneficiaries compared to those with private insurance with PAD, there is limited literature regarding whether a change in insurance coverage among Medicaid beneficiaries affects their care or outcomes [13,16].

This study aims to examine whether transitioning from Medicaid to commercial insurance affects the outcomes of patients with DFUs and lowers the risk of amputation. Understanding the impact of insurance coverage change on this vulnerable population is critical for developing effective strategies to improve health outcomes and reduce healthcare costs associated with DFUs.

## 2. Material and Methods

### 2.1. Dataset Background

This project used the PearlDiver Mariner database, a comprehensive all-payer (https://pearldiverinc.com (accessed on 30 August 2022)) [20–22], which collects insurance claims data from commercial payers, Medicaid/Medicare, other government payors, and cash pay claims. As of November 2021, the database contains data for over 122 million unique patients, covering the years 2010 to 2020. Patients are tracked over time, even when they move between states or switch payors. The analysis was completed on 22 April 2023 using the built-in statistical analysis software (Bellwether Research Software^™^) within the database. The Institutional Review Board (IRB) at the University of Arizona approved the study, and informed consent requirements were waived as the project used de-identified data (ID 00000155). The study followed the Strengthening the Reporting of Observational Studies in Epidemiology (STROBE) reporting guideline [23].

### 2.2. Study Variables

**Figure 1.**
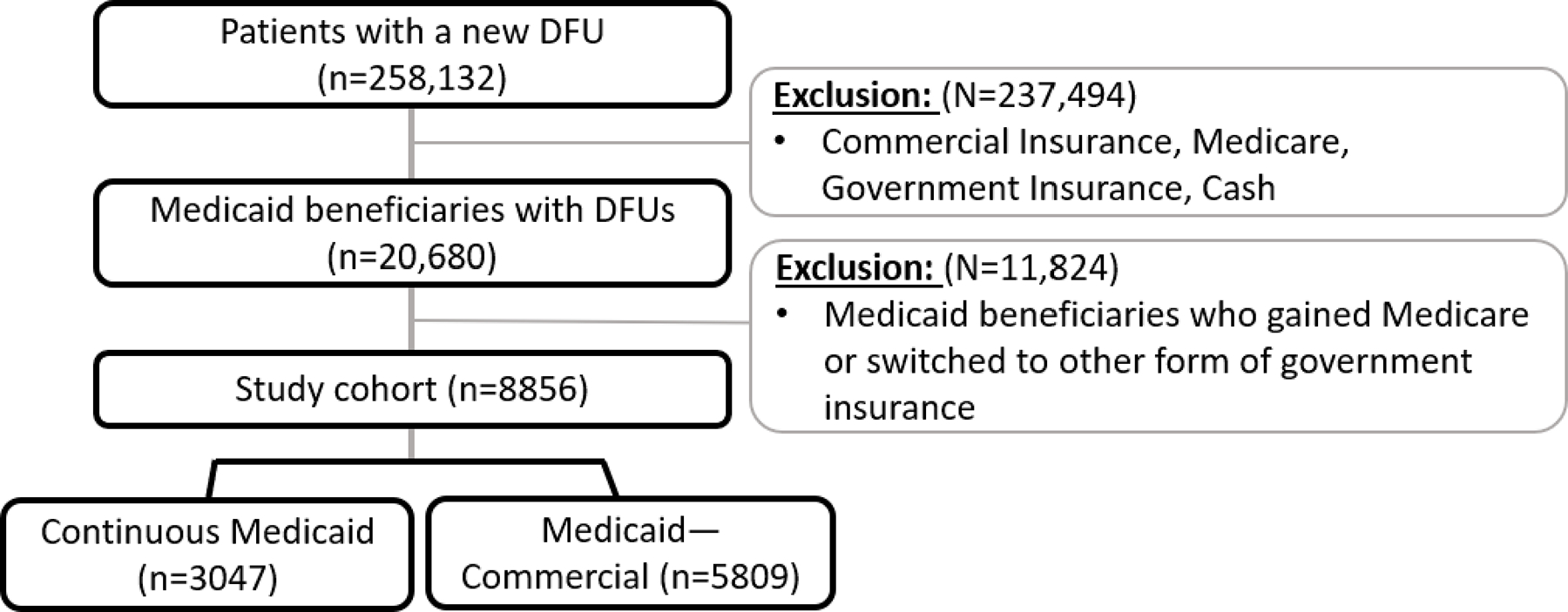
Study Flowchart.

The study cohort was identified using the International Classification of Diseases’ (ICD) 9 diagnosis codes. The database was queried for all adults over the age of 18 years with a diagnosis of DFU. Patients were included at the first appearance of the diagnosis code in their claims record. We excluded patients with a history of major amputation on either leg and those who were without continuous data for 6 months before enrollment and/or 36 months of continuous data after enrollment. Enrollment was rolling within the study period (2010–2020), requiring a total of 42 months of continuous data within this period. The initial study cohort comprised 20,680 Medicaid beneficiaries with DFUs. We further excluded patients who transitioned to Medicare or another government insurance (such as the Tricare program, n = 11,824), resulting in a final analysis cohort of 8856 Medicaid beneficiaries with DFUs. A detailed flowchart of the study cohort selection process is provided in Figure 1.

The primary outcome of our study was major amputation, defined as above, below, or through the knee amputation. The corresponding ICD-9 procedure codes were 84.10, 84.13–17, and 84.3, and used Current Procedural Terminology (CPT) codes of 27590–92, 27594, 27596, 27598, 27880–82, and 27884. Our secondary outcome was minor amputation, defined as amputation below the ankle, with ICD-9 procedure codes of 84.11–12 and CPT codes of 28800, 28805, 28810, 28820, and 28825.

The study cohort was divided into two groups: Medicaid beneficiaries with continuous Medicaid coverage for at least 12 months (continuous) and Medicaid beneficiaries who transitioned to commercial insurance coverage (Medicaid to commercial insurance). We assessed various patient demographic variables, including age, gender, and the Charlson Comorbidity Index (CCI) score, a validated tool that predicts survival in patients with multiple chronic illnesses such as diabetes and PAD. CCI score was categorized into 0–4 (mild to moderate) and 5 and above (severe). In addition, we evaluated patient comorbidities such as chronic obstructive pulmonary disease (COPD), chronic kidney disease (CKD), end-stage renal disease (ESRD), cerebrovascular disease, coronary artery disease, congestive heart failure, liver disease, obesity, hypertension, hyperlipidemia, depression, and tobacco abuse.

### 2.3. Statistical Analysis Methods

The outcomes of patients with DFUs in two groups, continuous and Medicaid to commercial insurance, were compared. Baseline demographics and characteristics were compared. To estimate major amputation-free survival, Kaplan–Meier estimate curves were generated. For time-to-event analysis, the Kaplan–Meier estimate was used instead of univariate logistic regression because it is non-parametric and can handle censored data. Additionally, we performed propensity score matching, whereby each patient in the continuous group was matched to one patient in the Medicaid to commercial insurance group using their age, gender, and CCI (1:1 ratio). The “nearest neighbor” matching method was used to create a propensity-matched cohort. A propensity score was calculated for each beneficiary. Nearest neighbor matching paired each beneficiary in the continuous group with a beneficiary in the Medicaid to commercial insurance group with the closest propensity score. To ensure an adequate sample size in the propensity-matched cohort, we have limited the covariates used to calculate the propensity score to age, gender, and CCI. We started by conducting univariate analyses to determine the rates for major and minor amputation. Logistic regression models were performed to conduct multivariable analyses on both the entire study cohort and the propensity-matched cohort. The significance level was set to a *p*-value of 0.05.

## 3. Results

There were 8856 Medicaid beneficiaries with DFUs included in the study cohort. Of these, 3047 patients (34.6%) were in the continuous group, and 5809 (66.4%) were in the Medicaid to commercial insurance group. Patients in the continuous group were more likely to be male and older compared to their counterparts in the Medicaid to commercial insurance group, which had a higher CCI score. Additionally, patients in the Medicaid to commercial insurance group had a higher rate of obesity, depression, smoking, and COPD. In contrast, patients in the continuous group had a higher rate of kidney disease. Refer to Table 1 for detailed information.

The overall rate of major amputation was 2.8% (n = 247), and it was significantly higher for the continuous group than the Medicaid to commercial insurance group (n = 96, 3.2% vs. n = 151, 2.6%, *p* < 0.05). The overall minor amputation rate was 4.9%, and it was higher for the continuous group (5.4% vs. 4.7%, *p* < 0.05). The average duration of continuous follow-up for patients in the study cohort was 3.8 years.

**Figure 2.**
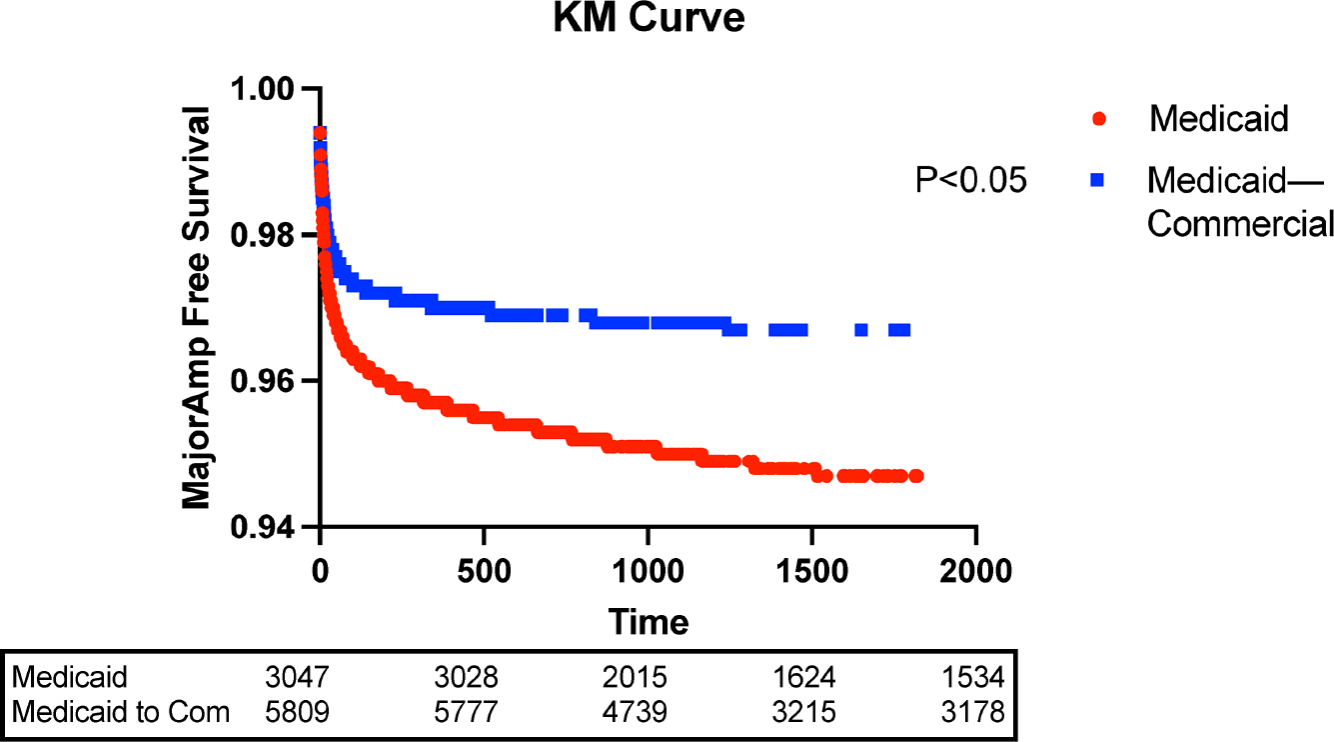
Kaplan–Meier major amputation-free survival.

The Kaplan–Meier curves demonstrated that the major amputation-free survival was higher for the Medicaid to commercial insurance group than the continuous group (96.7% vs. 94.7%, *p* < 0.05) (see Figure 2). In the multivariate analysis, Medicaid beneficiaries in the Medicaid to commercial insurance group had a 27% lower risk of major amputation (OR 0.73, 95% CI 0.55–0.97, *p* < 0.05) than the continuous group. However, the two groups had no significant difference in the risk of minor amputation (OR 0.92, 95% CI 0.75–1.14, *p* > 0.05). Other factors, including high CCI, male gender, chronic kidney disease, and PAD, contributed to an increased risk of major amputation (refer to Table 2).

### Propensity-Matched Cohort

**Figure 3.**
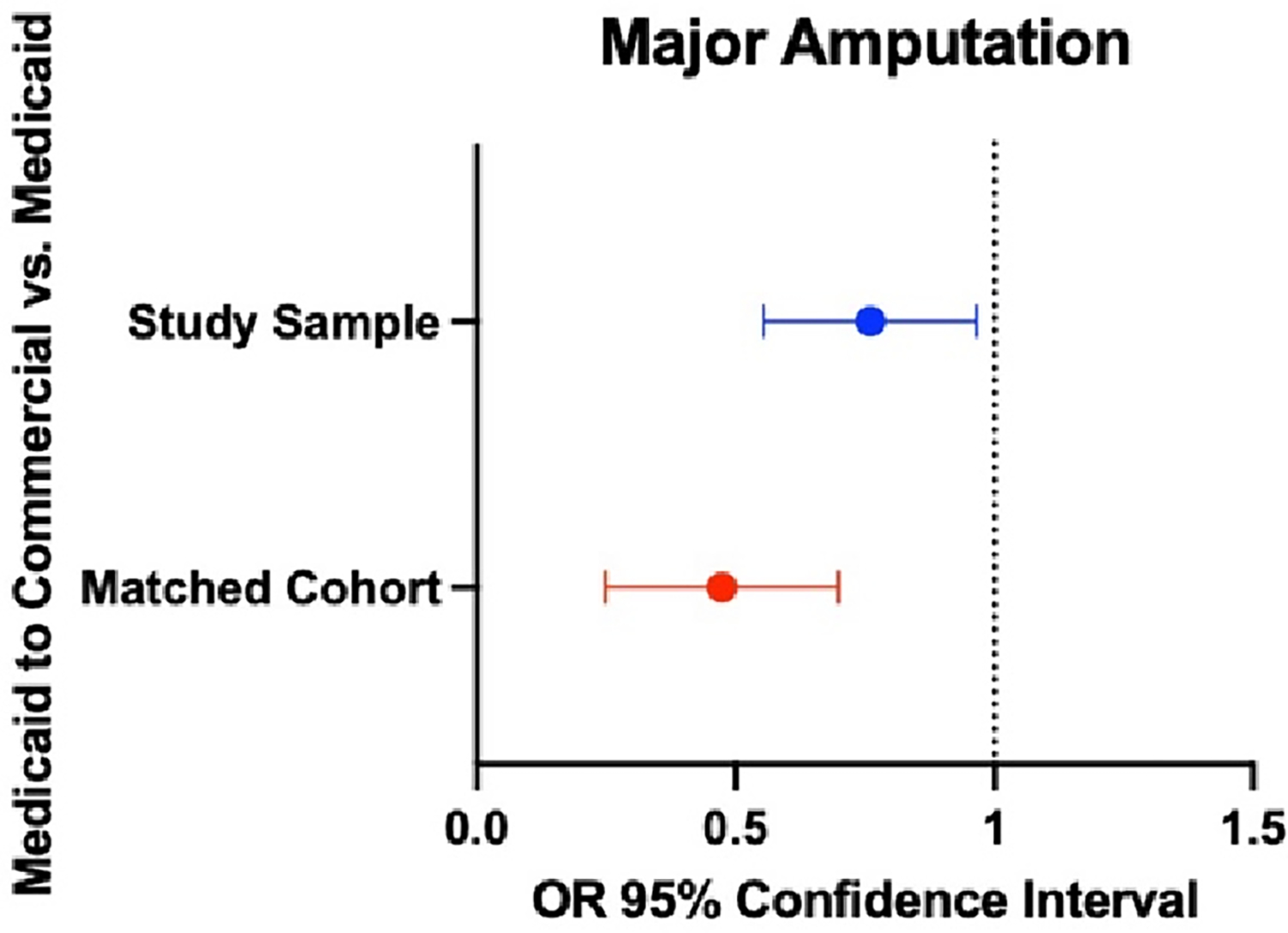
Multivariable analysis results. Blue dot, risk of major amputation risk for study cohort; Red dot, risk of major amputation for propensity matched cohort.

The propensity-matched cohort included 1509 Medicaid beneficiaries with DFUs in both the continuous Medicaid group and the Medicaid to commercial insurance group (refer to Table 3). Multivariable analysis revealed that the Medicaid to commercial insurance group had a 35% lower risk of major amputation (OR 0.65, 95% 0.22, 0.55, *p* = 0.01) compared to those in the continuous group (Figure 3).

## 4. Discussion

In our retrospective review of a comprehensive insurance claims database, we found a significant link between changes in insurance coverage and outcomes among Medicaid beneficiaries with DFUs. Our results demonstrated that Medicaid beneficiaries who transitioned from Medicaid to commercial insurance had a 27% lower risk of major amputation than those with continuous Medicaid coverage. These findings were further confirmed in the propensity-matched cohort.

Previous research has explored the relationship between health insurance status and the care and outcomes of patients with vascular disease. Giacovelli et al. found that patients with Medicaid or no health insurance had more severe vascular disease at the time of presentation, with uninsured patients and Medicaid beneficiaries having a significantly high risk of presenting with ruptured abdominal aortic aneurysms and symptomatic carotid artery stenosis than insured, non-Medicaid patients [24]. Similarly, Medicaid beneficiaries and uninsured patients with PAD were more likely to present with chronic limb-threatening ischemia than claudication compared to those with commercial insurance [24]. A study by Kim et al. found that patients with Medicare and Medicaid had a higher number of comorbidities, presented with more advanced stages of PAD, and were more likely to undergo amputation compared to those with private insurance or covered under a Health Maintenance Organization (HMO) [25]. The association between insurance status and vascular disease outcomes could be influenced by factors such as socioeconomic characteristics, environmental factors, and healthcare system characteristics [26–29].

One possible explanation for the association between insurance type and outcomes is the different populations covered by Medicaid and commercial insurance. Medicaid is a public health insurance that primarily covers low-income individuals, including a significant proportion of racial and ethnic minority adults [26,30]. Additionally, Medicaid reimbursement rates for providers are generally lower than commercial insurance rates, with Medicaid fee-for-service payments for physician services being nearly 30% below Medicare payments and even lower than commercial insurance payments [31]. This may lead to reduced provider participation and potentially limited access to primary care for DFU care, particularly among low socioeconomic populations. In times of workforce shortage, access the healthcare services can be further constrained.

Mixed findings exist regarding the accessibility and quality of healthcare offered by publicly sponsored insurance programs and commercial insurance. According to Wray et al., individuals with commercial insurance are less likely to face difficulty seeing a physician and being unable to afford medication [32]. These variations in findings can be attributed to the differing benefits of Medicaid programs in some states. Although Medicaid expansion has benefited individuals with DFUs and without health insurance, access to primary and specialty care services still poses challenges when compared to commercial health insurance [18,19,32]. Furthermore, the coverage of optional services such as podiatry care may be restricted for Medicaid beneficiaries in some states compared to those with commercial health insurance and Medicare, which is relevant to DFU care [33–35].

The limitations of the study include: 1. Our study used a retrospective review of a claim-based dataset, recognizing the biases associated with observational data. The database does not include information on race, ethnicity, or the severity of DFUs, which could impact DFU-related outcomes. Covariates such as the intersectionality of gender and race, and the influence of low-income and rural neighborhoods, can amplify the risk of limb loss, but we were unable to analyze these due to the limitations of the dataset. 2. We are unaware of the exact reasons why patients changed from Medicaid to commercial insurance coverage, although it could be due to improvements in employment status and/or socioeconomic status. 3. We are unable to evaluate whether there is a difference in care quality among Medicaid beneficiaries with DFUs between the continuous group and the Medicaid to commercial insurance group, including but not limited to better access to primary care and podiatry care. 4. The reasons for the differences in comorbidities between the continuous Medicaid and Medicaid to commercial insurance groups are unclear. The continuous Medicaid group was more likely to have CKD and ESRD, while the Medicaid to commercial group had higher percentages of COPD, obesity, depression, and smoking. It is possible that providers who care for patients with commercial insurance may code for these comorbidities differently or more frequently than those caring for Medicaid beneficiaries, but this could not be determined due to the limitations of the dataset. 5. We did not perform any assessment of the propensity matching quality due to limitations of the built-in statistical analysis software. Although our study has the limitations mentioned above, we were able to analyze a large cohort of Medicaid beneficiaries with DFUs and found that transitioning from Medicaid to commercial insurance is an independent risk modifier of major lower extremity amputation. Our study is the first to demonstrate that Medicaid beneficiaries who switched to commercial insurance had a significantly lower risk of major amputation than those with continuous Medicaid coverage.

## 5. Conclusions

In conclusion, this study shows an association between changes in insurance coverage and outcomes for Medicaid beneficiaries with DFUs. Our results indicate that patients who switched from Medicaid to commercial insurance had a substantially lower risk of major amputation compared to those who remained on Medicaid coverage. These results show the need for further research to evaluate the impact of health insurance coverage on the care and outcomes of low-income populations at risk of limb loss.

## Figures and Tables

**Table 1.** Characteristics and demographics of the study cohort.

	Continuous Medicaid n = 3047		Medicaid to Commercial Insurance n = 5809		Standard Mean Difference	*p*-Value

	n	%	n	%		

Demographic						
Age,y					0.56	<0.001
<40	664	21.8	1709	29.4		
40–49	747	24.5	1519	26.1		
50–59	1171	38.4	1907	32.8		
60–69	373	12.2	542	9.3		
≥70	75	2.5	48	0.8		

Gender					0.05	0.03
Male	1431	47.0	2587	44.5		
Female	1616	53.0	3222	55.5		

CCI					0.3	<0.001
0–4	2949	96.8	5432	93.5		
≥5	73	3.2	355	6.1		
Comorbidities						
COPD	1556	51.0	3370	58.0	0.29	<0.001
CVD	807	26.5	1582	27.2	0.11	0.47
CHF	573	18.8	1085	18.7	0.07	0.90
CAD	1012	33.2	1960	33.7	0.14	0.63
HTN	2631	86.3	5062	87.1	0.16	0.31
CKD	988	32.4	1549	26.7	0.09	<0.001
ESRD	631	20.7	961	16.5	0.05	<0.001
PAD	1112	36.5	2117	36.4	0.22	0.98
Obesity	1553	51.0	3733	64.2	0.33	<0.001
Depression	1569	51.5	3703	63.7	0.10	<0.001
Smoking	1681	55.2	3985	68.6	0.34	<0.001

CCI, Charlson Comorbidity Index; COPD, chronic obstructive pulmonary disease; CVD, cerebrovascular disease; CHF, congestive heart failure; CAD, coronary artery disease; HTN, hypertension; CKD, chronic kidney disease; ESRD, end-stage renal disease; PAD, peripheral artery disease.

**Table 2.** Multivariable analysis for major amputation in the study cohort.

	Odds Ratios	95% Confidence Interval	*p*-Value
Medicaid to commercial insurance	0.73	0.56–0.97	0.02
Age	0.99	0.97–1.00	0.05
Charlson Comorbidity Index (CCI)	1.03	0.97–1.00	0.37
Male gender	1.48	1.13–1.95	0.004
Cerebrovascular disease	1.28	0.97–1.68	0.08
Chronic kidney disease	1.52	1.07–2.13	0.017
Congestive heart failure	1.03	0.75–1.41	0.84
Coronary artery disease	1.49	1.11–2.01	0.008
Hypertension	3.76	1.53–12.5	0.01
Depression	1.52	1.14–2.04	0.004
Peripheral artery disease	7.14	4.96–10.5	<0.001
Renal failure	1.15	0.80–1.66	0.46
Smoking	1.57	1.14–2.18	0.006

**Table 3.** Characteristics and demographics of the propensity-matched cohort.

	Continuous Medicaid n = 1509		Medicaid to Commercial Insurance n = 1509		*p*-Value

	n	%	n	%	

Age,y					1.0
<40	324	21.5	321	21.3	
40–49	374	24.8	379	25.1	
50–59	587	38.9	586	38.8	
60–69	184	12.2	186	12.3	

Gender					0.99
Male	715	47.4	717	47.5	
Female	794	52.6	792	52.5	

CCI					0.99
0–4	1452	96.2	1449	96.0	
≥5	57	3.8	60	4.0	

CKD	398	26.4	395	26.2	0.99
CAD	506	33.5	539	35.7	0.99
PAD	545	36.1	558	37.0	0.76
Smoking	828	54.9	815	54.0	0.80

CCI, Charlson Comorbidity Index; CAD coronary artery disease; CKD, chronic kidney disease; PAD, peripheral artery disease.

## Data Availability

The data underlying this study belong to PearlDiver Inc. The authors conducted data analysis using the build-in Bellwether Research Software^™^. Data are available to researcher through a subscription program. Future researchers interested in using the data can subscripe to the research program. Further instructions on the program can be found at https://pearldiverinc.com.
